# Risk factor *SORL1*: from genetic association to functional validation in Alzheimer’s disease

**DOI:** 10.1007/s00401-016-1615-4

**Published:** 2016-09-16

**Authors:** Olav M. Andersen, Ina-Maria Rudolph, Thomas E. Willnow

**Affiliations:** 1 Department of Biomedicine, Danish Research Institute of Translational Neuroscience DANDRITE-Nordic EMBL Partnership for Molecular Medicine, Aarhus University, Ole Worms Alle 3, Aarhus C, 8000 Aarhus, Denmark; 2 Max-Delbrueck-Center for Molecular Medicine, Robert-Roessle-Strasse 10, 13125 Berlin, Germany

## Abstract

Alzheimer’s disease (AD) represents one of the most dramatic threats to healthy aging and devising effective treatments for this devastating condition remains a major challenge in biomedical research. Much has been learned about the molecular concepts that govern proteolytic processing of the amyloid precursor protein to amyloid-β peptides (Aβ), and how accelerated accumulation of neurotoxic Aβ peptides underlies neuronal cell death in rare familial but also common sporadic forms of this disease. Out of a plethora of proposed modulators of amyloidogenic processing, one protein emerged as a key factor in AD pathology, a neuronal sorting receptor termed SORLA. Independent approaches using human genetics, clinical pathology, or exploratory studies in animal models all converge on this receptor that is now considered a central player in AD-related processes by many. This review will provide a comprehensive overview of the evidence implicating SORLA-mediated protein sorting in neurodegenerative processes, and how receptor gene variants in the human population impair functional receptor expression in sporadic but possibly also in autosomal-dominant forms of AD.

## Introduction

Undoubtedly, Alzheimer’s disease (AD) represents one of the most dramatic threats to healthy aging in all societies and devising effective treatments for this devastating condition remains a major challenge in biomedical research. Much has been learned about the molecular concepts that govern proteolytic processing of the amyloid precursor protein (APP) to amyloid-β peptides (Aβ), and how accelerated accumulation of neurotoxic Aβ peptides causes neurodegeneration in rare familial but also common sporadic forms of AD (see “Box” for details). Targeting the accumulation of Aβ in the brain of patients holds great promise for success in the clinics. However, currently, this approach is limited to few targets, such as β- and γ-secretases, the enzymes that breakdown APP to Aβ [18, 21]. Thus, major efforts have been undertaken in recent years to identify additional players in Aβ metabolism and action, and to validate their relevance as therapeutic targets in treatment of AD.

Out of a plethora of proposed modulators of APP processing, one protein emerges as a promising candidate in AD pathology, a sorting receptor called sorting-related receptor with A-type repeats (SORLA) (also known as SORL1 or LR11). Independent approaches using human genetics, clinical pathology, or functional studies in animal models all converge on this receptor that is now considered an important factor in AD-related processes by many. This review provides a timely overview of the evidence implicating SORLA in AD. We describe the association of *SORL1*, the gene encoding SORLA, with the occurrence of sporadic but also autosomal-dominant forms of AD. We detail studies in cell and animal models that identified the molecular mechanism of SORLA as neuronal sorting receptor in control of amyloidogenic processes in the brain. We discuss the functional implications of sequence variations in *SORL1* found in individuals with AD, and we review (pre)clinical data that explore the predictive value of SORLA levels in assessment of risk and outcome of AD, and that document the therapeutic benefit of strengthening receptor activity in treatment of AD-related conditions.

## Box: The amyloid cascade hypothesis

The amyloid cascade hypothesis represents a widely accepted concept to describe the cellular events underlying neurodegenerative processes in AD [34, 88, 89]. Central to this hypothesis is the amyloid precursor protein (APP), a 110–130 kDa type-1 transmembrane protein expressed in three major isoforms APP_695_, APP_751_, and APP_770_. All APP variants (including neuronal APP_695_) share a peptide sequence as part of their transmembrane and extracellular domains called the Aβ peptide. In a natural process occurring in many cell types, APP undergoes two alternative processing pathways [88]. In one pathway (figure panel a, to the right), APP is cleaved by a protease activity called α-secretase that produces soluble (s) APPα and a membrane-anchored fragment CTFα. Subsequently, the multimeric γ-secretase complex cleaves CTFα into peptide P3 and the APP intracellular domain (AICD) [49]. Because α-secretase cleavage destroys the Aβ peptide, this pathway acts non-amyloidogenic. In contrast, the disease-promoting (amyloidogenic) pathway is initiated by the cleavage of APP by β-secretase at the amino terminal end of Aβ, followed by γ-secretase cleavage at its carboxyl terminus [14, 94]. These steps produce Aβ peptides of mainly 40–42 amino acids length, as well as sAPPβ and the AICD (figure panel a, to the left). Recently, a novel secretase activity, termed η-secretase, has been identified that also acts on the APP precursor polypeptide (panel b in the figure) [99]. This protease produces a carboxyl terminal stub CTFη that serves as alternative substrate to α- and β-secretases in non-amyloidogenic and amyloidogenic processing, respectively. Evidence that the extent of breakdown of APP to Aβ determines onset and progression of AD stems from rare autosomal dominant, early onset forms of AD caused by mutations in the genes encoding APP or in presenilin-1 or -2 (*PSEN1*, *PSEN2*), subunits of the γ-secretase complex. These mutations are typically associated with an overall increase in the production of Aβ or with a shift towards generation of the more disease-prone variant Aβ42 [45]. Although the causal role of Aβ in AD is undisputed, its mode of action is still a matter of investigation. According to current hypotheses, soluble oligomeric forms of Aβ act as physiological modulators of synaptic activity and aberrant suppression of synaptic transmission, caused by excessive Aβ accumulation, is responsible for synaptic dysfunction and eventual neuronal cell death in the AD brain [96]. Similar to rare early onset AD, the more common sporadic or late-onset form of AD (>95 % of cases) also has a strong genetic component. Many risk genes have been identified that promote onset and progression of late-onset AD, chief among which is the gene for apolipoprotein (APO) E, a lipid transporter in the brain [19, 91]. Conceptually, risk factors for sporadic AD may work via numerous mechanisms to aggravate neurodegenerative processes. Still, many of them are believed to also act through enhancing the accumulation and neurotoxic action of Aβ. 
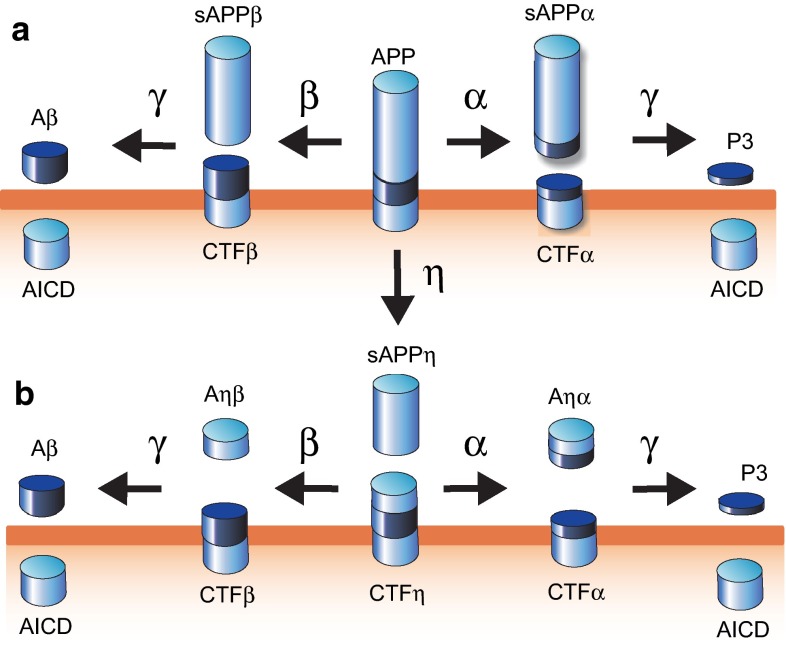


## SORLA, a neuronal sorting receptor in APP processing

**Fig. 1:**
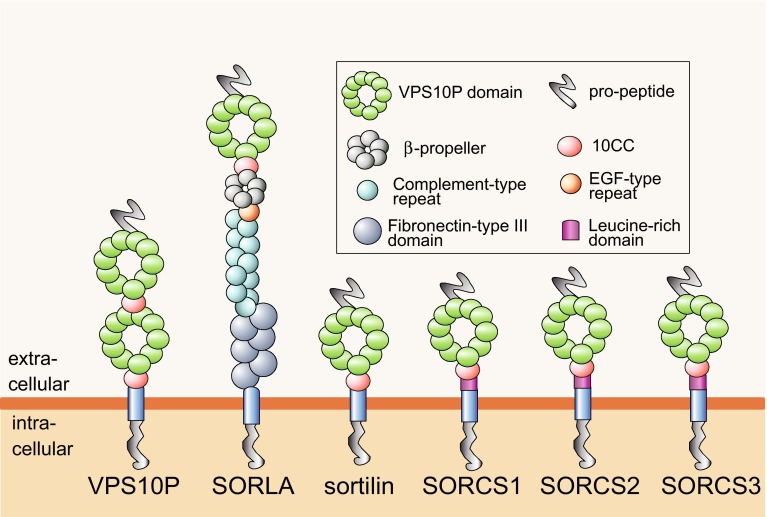
SORLA, a member of the VPS10P domain receptor gene family of neuronal sorting receptors. Sorting-related receptor with A-type repeats (SORLA) is member of the vacuolar protein sorting (VPS10P) domain receptor gene family, a group of five related type-1 transmembrane proteins found in mammalian cell types [101]. Other family members are sortilin as well as sorting receptor CNS expressed (SORCS) 1, SORCS2, and SORCS3. All receptors share an extracellular VPS10P domain, a single transmembrane domain, and a short cytoplasmic tail. The receptors are produced as precursor proteins containing a short pro-peptide at the amino terminus that blocks ligand binding in the VPS10P domain. Proteolytic processing of the pro-peptide by convertases in the Golgi is a precondition for activating the ligand-binding capability of the receptors [41]. SORLA is unique among the members of the VPS10P domain receptor gene family as it contains additional functional modules not shared by the other receptors including domains for protein–protein interaction (fibronectin-type III domains, complement-type repeats) or for pH-dependent release of ligands in endosomes (6-bladed β-propeller). Complement-type repeats and the β-propeller are functional elements also found in lipoprotein receptors, such as the low-density lipoprotein receptor, suggesting the possibility of SORLA to act in cellular lipoprotein transport [80]

The first evidence implicating SORLA in AD came from a study by Scherzer and colleagues who performed global gene expression profiling to identify genes differentially expressed in lymphoblasts and in brain autopsies from AD patients compared to control subjects. This study identified a 2.5-fold decrease in brain SORLA levels in some sporadic cases of AD. Loss of protein expression was seen in cortex and hippocampus, but not in the cerebellum of affected individuals [83]. SORLA is a 250 kDa transmembrane protein that was identified prior in a quest for novel lipoprotein receptors expressed in the mammalian brain [42, 102]. Although SORLA showed some structural resemblance to lipoprotein receptors, a novel structural element not seen in any mammalian protein before was most noteworthy (Fig. 1). This so-called VPS10P domain is a 700 amino acid module in the extracellular domain of the receptor that folds into a ten-bladed β-propeller and that represents a binding site for peptide ligands [46, 70]. The VPS10P domain had been identified initially in the vacuolar protein sorting 10 protein (VPS10P), a sorting factor in yeast that directs target proteins from the Golgi to lysosomal compartments [58]. Today, this domain is the unifying structural motif of a group of five related VPS10P domain receptors that act in intracellular sorting processes in neuronal and multiple non-neuronal cell types in the mammalian organism (reviewed in [101]) (Fig. 1).

Abundant expression of SORLA was seen in neurons throughout the central nervous system including cortex, hippocampus, cerebellum, and spinal cord [42, 102]. In neurons, SORLA mainly localized to intracellular compartments in the cell soma, suggesting a role for this receptor in vesicular protein transport [63]. Based on this assumption, two subsequent studies proposed a molecular concept whereby SORLA acts as a sorting factor for APP, guiding intracellular trafficking and processing of this precursor protein [4, 68]. In these studies, overexpression of SORLA in cell lines reduced [4, 68] while loss of expression in gene-targeted mice increased Aβ production [4], providing an explanatory model for why reduced *SORL1* expression in some individuals with sporadic AD may promote neurodegeneration.

## *SORL1* is genetically implicated in late- and early onset forms of AD

Initial data on the role of SORLA in AD were met with considerable skepticism as SORLA appeared as one of many proposed modulators of APP processing. However, strong support for a causal involvement of this receptor in neurodegenerative disease came with genetic studies associating *SORL1* gene variants with the occurrence of sporadic AD. In a pioneering study, Rogaeva et al. used a candidate gene approach to document association of inherited variants in *SORL1* with sporadic AD in Caucasians [76]. This finding was replicated in some association studies, while others failed to confirm it (summarized in [74]). This controversy was attributed to allelic heterogeneity in various ethnicities and to the lack of statistical power due to small cohort sizes. Ultimately, this discrepancy was resolved by combining the findings of many studies in meta-analyses substantiating the association of *SORL1* variants with sporadic AD [44, 74, 97]. Recently, genome-wide association studies (GWAS) confirmed association of *SORL1* with the sporadic late-onset form of AD in populations of Caucasian and Asian origin [51, 62].

**Fig. 2:**
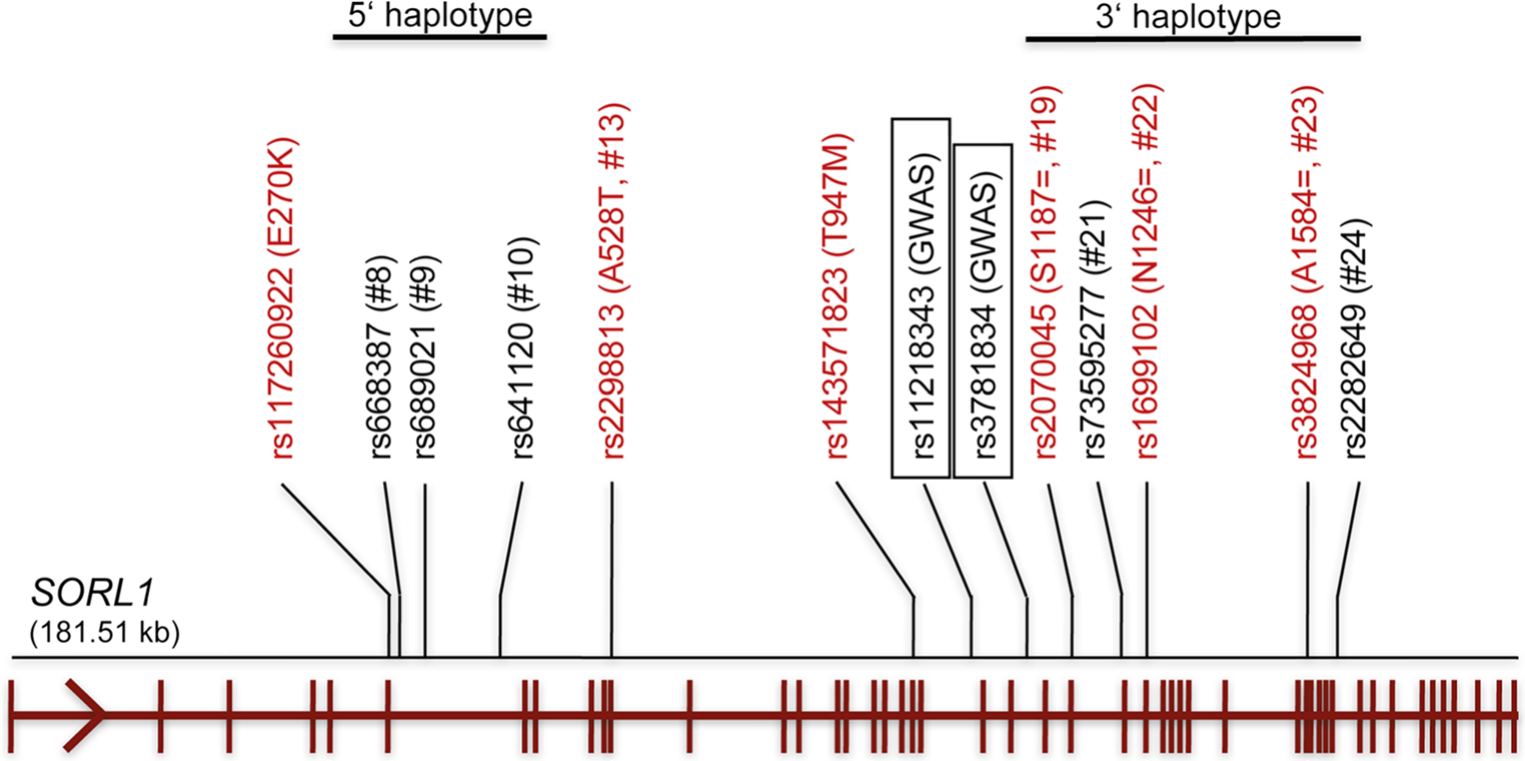
Single nucleotide polymorphisms in *SORL1* associated with sporadic AD. Selected coding (*red*) and non-coding (*black*) single nucleotide polymorphisms (SNPs) in human *SORL1* associated with sporadic AD are indicated. The numbering of the SNPs follows the nomenclature introduced by Rogaeva and colleagues [76]. Most SNPs cluster in two haplotype blocks in the 5′ and 3′ region of the gene locus. SNPs rs11218343 and rs3781834 are associated with sporadic AD at a genome-wide level in individuals of Caucasian and Asian origin [51, 62]. The structure of *SORL1* on chromosome 11q23-q24 is indicated below with exons represented by vertical lines. For coding variants, the change in amino acid sequence is given in *brackets* [12]

Taken together, a number of single nucleotide polymorphisms (SNPs) in *SORL1* have been associated with the occurrence of sporadic AD. These SNPs include coding as well as non-coding sequence variations and cluster in two haplotype blocks in the 5′ and 3′ regions of the gene on human chromosome 11q23-q24 (see Fig. 2 for numbering of the SNPs). Refined clinical analyses document association of individual SNPs with distinct neuropathological features including deposition of senile plaques (SNP#8) and fibrillary tangles (SNP#10, rs11218343) [9, 25], and with loss of gray matter volume (SNP#23) [39] and hippocampal atrophy (SNP#21-26) [20]. SNPs in the 3′ haplotype block are associated with pathological alterations of AD biomarkers in cerebrospinal fluid (CSF), such as Aβ and tau [2, 24, 31, 47, 55], whereas SNP#8 [6], SNP#19 [75], and rs11218343 [64] predict longitudinal cognitive change. Interestingly, SNP#19 appears to have a gender bias impacting cognitive decline stronger in females than in males [75].

An exciting twist in the genetic of *SORL1* came with the observation that sequence variations in this gene are also found in individuals suffering from autosomal-dominant forms of AD, rendering SORLA a potential culprit in both late- and early onset types of this disease [65, 69, 95]. So far, mutations in three genes have been shown to cause the rare early onset form of AD, namely *APP*, *PSEN1* and *PSEN2*. However, the majority of individuals suffering from early onset AD lack obvious mutations in *APP*, *PSEN1,* and *PSEN2*, indicating the existence of additional genes that cause this aggressive form of neurodegeneration [30]. Using whole exome sequencing or candidate gene approaches, coding sequence variations in *SORL1* have been identified in early onset cases of AD in which inheritance was consistent with autosomal-dominant transmission. Identified sequence variants in *SORL1* include nonsense and frame shift as well as potentially damaging missense alterations [65, 69, 95]. Many of these coding variants were not seen in control subjects as exemplified in the study by Nicolas and colleagues [65] that identified a total of 50 rare missense variants (minor allele frequency <1 %), with 37 variants in AD cases but only 17 in control subjects. These data reached exome-wide significance when the analysis was restricted to a subset of patients with a positive family history. These observations still need to be interpreted with some caution as documentation of cosegregation of these variants in affected relatives has so far only been shown for two cases, p.G511R [69] and p.Y1816C [95]. However, if substantiated in further studies, these findings suggest *SORL1* as a novel disease gene in autosomal-dominant forms of AD, lending further support to the central role of amyloidogenic processing in the etiology of this disorder.

## SORLA, an inhibitor of amyloid-β peptide accumulation in the brain

**Fig. 3:**
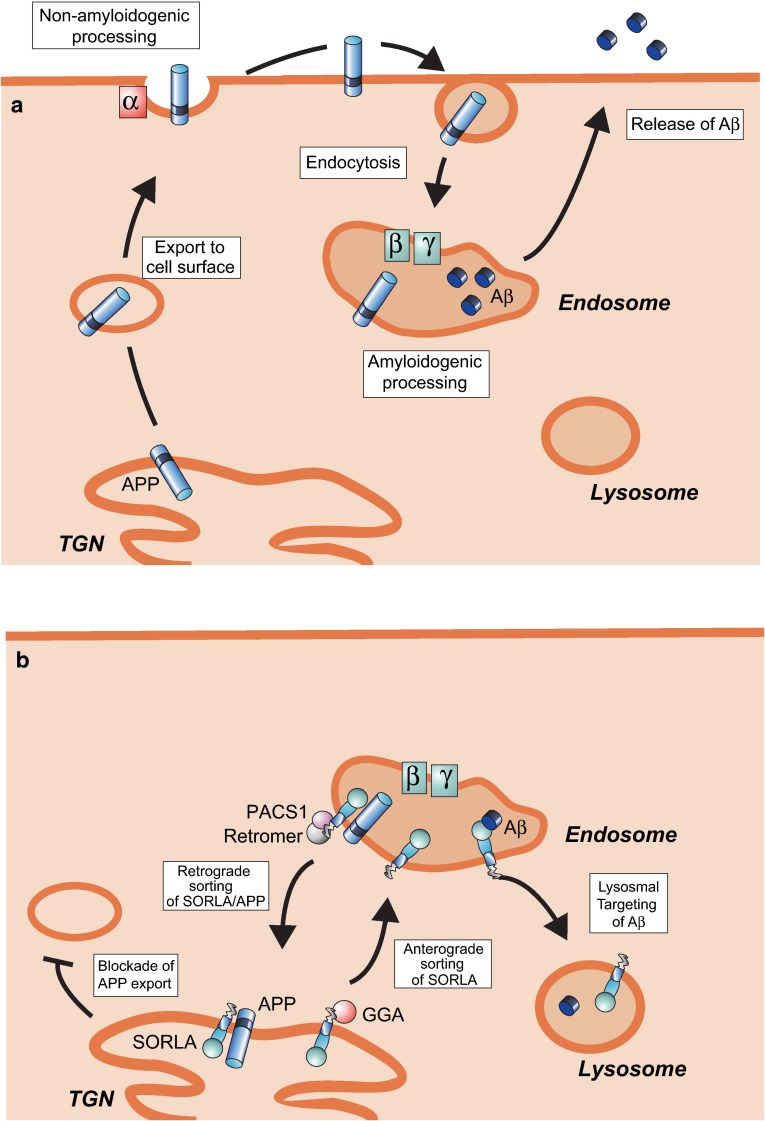
SORLA-dependent sorting of APP and Aβ. **a** Nascent APP molecules move through the *trans*-Golgi-network (TGN) to the cell surface where they are subject to non-amyloidogenic processing initiated by α-secretase (α) cleavage. APP molecules not cleaved by α-secretase at the cell surface undergo endocytosis and move to endosomes where they are processed by β- and γ-secretases (β, γ) producing Aβ. Aβ peptides are released from cells through various exocytic mechanisms. **b** SORLA acts as a sorting receptor for APP causing retrograde endosome to TGN retrieval and slowing down exit from the Golgi to reduce the number of APP molecules subjected to amyloidogenic processing. SORLA also acts as anterograde sorting factor that directs newly produced Aβ molecules to lysosomal degradation, further decreasing the amyloidogenic burden. Anterograde sorting also results in lysosomal breakdown of SORLA, negatively regulating receptor levels [23]. Some cytosolic adaptors required for shuttling of SORLA between TGN and endosomes are indicated and include GGAs (Golgi-localizing, y-adaptin ear homology domain, ARF-interacting proteins) for anterograde as well as retromer and PACS1 (phosphofurin acidic cluster sorting protein 1) for retrograde sorting. Figures adapted from [100]

What is the mechanism whereby SORLA acts as a risk factor in AD? This question has initially been addressed in cell lines (summarized in [100]), and recently also substantiated in transgenic mouse models, identifying SORLA’s mode of action as a neuronal sorting receptor for APP and Aβ. The basis for its action is the complex trafficking path whereby APP moves between intracellular compartments and the cell surface, determining the extent of Aβ accumulation (reviewed in [81]). In a simplified scheme (Fig. 3a), newly synthesized APP molecules traffic through the secretory pathway to the cell surface. In route, they encounter α-secretase, resulting in non-amyloidogenic cleavage (see also “Box”). Precursor molecules escaping non-amyloidogenic processing are internalized from the cell surface into endosomes. In endocytic compartments, APP is processed by β- and γ-secretases producing Aβ [28, 32, 72, 82]. Intracellular accumulation [8] as well as secretion of Aβ peptides, involving exosomes [71] and other means of exocytosis [7], contributes to the amyloidogenic burden in the brain. As it turns out, SORLA impairs amyloidogenic processes in two ways, both of which involve its ability to shuttle target proteins between secretory and endocytic compartments of the cell [41, 66, 85]. In one mechanism, SORLA acts as a sorting factor for APP retrogradely moving internalized precursors from early endosomes back to the *trans*-Golgi network (TGN) and slowing down exit from the Golgi, thereby reducing the number of APP molecules subjected to amyloidogenic processing (Fig. 3b). Consequently, overexpression of SORLA in neuronal and non-neuronal cell lines blocks APP processing and reduces Aβ production [4, 68, 76], while loss of SORLA increases Aβ levels and senile plaque burden in several mouse models of AD [4, 22, 77]. The second mode of receptor action involves the anterograde sorting of SORLA from the TGN to endosomes. This pathway not only serves to replenish receptor levels in endosomal compartments for APP retrieval, but it also results in lysosomal targeting of Aβ molecules that have been identified as another receptor ligand (Fig. 3b) [13].

Jointly, retrograde sorting of APP to the TGN and anterograde movement of Aβ to lysosomes reduce brain levels of Aβ and contribute to the protective action of SORLA in the brain. Its pathological relevance has been confirmed in unbiased siRNA screens that also identified this receptor as a major determining factor of Aβ levels in cells [15]. The quantitative contribution of either activity to the overall protective function of SORLA is difficult to assess. Based on mathematic models, the kinetic of SORLA and APP interaction appears as a major determinant of Aβ levels, arguing for a predominant role of the APP sorting pathway in defining the risk of AD [1, 53, 84]. In the healthy brain cortex, SORLA and APP are expressed in almost equimolar ratio, suggesting near complete saturation of APP molecules with sorting receptors [84]. Thus, reduced levels of SORLA, as in some individuals with sporadic AD, likely act through loss of protection of APP from processing.

## Molecular and structural basis of SORLA action

Two protein modules in SORLA define its function as a neuronal sorting receptor in AD, namely binding sites for APP and Aβ in the extracellular region as well as recognition motifs for cytosolic adaptors in the receptor tail that govern intracellular trafficking. Cell and structural biology approaches have provided an in-depth view of the structural basis of these domains for receptor function.

The binding site for APP in SORLA has been mapped to the cluster of eleven complement-type repeats in the extracellular domain of the receptor that forms a 1:1 stoichiometric complex with a region in the extracellular domain of APP referred to as the carbohydrate-linked domain [4, 5]. Deleting this cluster of complement-type repeats abolishes the ability of SORLA to protect APP from processing [61]. A second site of interaction may involve the cytosolic domains of both proteins as shown by fluorescence life-time imaging microscopy [90] and by mutagenesis of the APP tail [50]. The interaction of SORLA and APP is blocked by signaling through β-adrenergic receptors via a yet unknown mechanism, resulting in impaired Golgi retrieval and in increased endosomal accumulation of APP [16]. The binding site for Aβ in SORLA has been mapped to the VPS10P domain using X-ray crystallography [46]. Disruption of this binding site reduces lysosomal catabolism of Aβ without impacting APP processing rates, suggesting that sorting of Aβ and APP is two distinct receptor functions [13]. The complement-type repeats are a feature of SORLA not shared by other VPS10P domain receptors (see Fig. 2). Also, Aβ binds to the VSP10P domain of SORLA but not to the closely related domain in sortilin [13], arguing for a unique role of SORLA among the members of the VPS10P domain gene family in control of amyloidogenic processing.

**Fig. 4:**
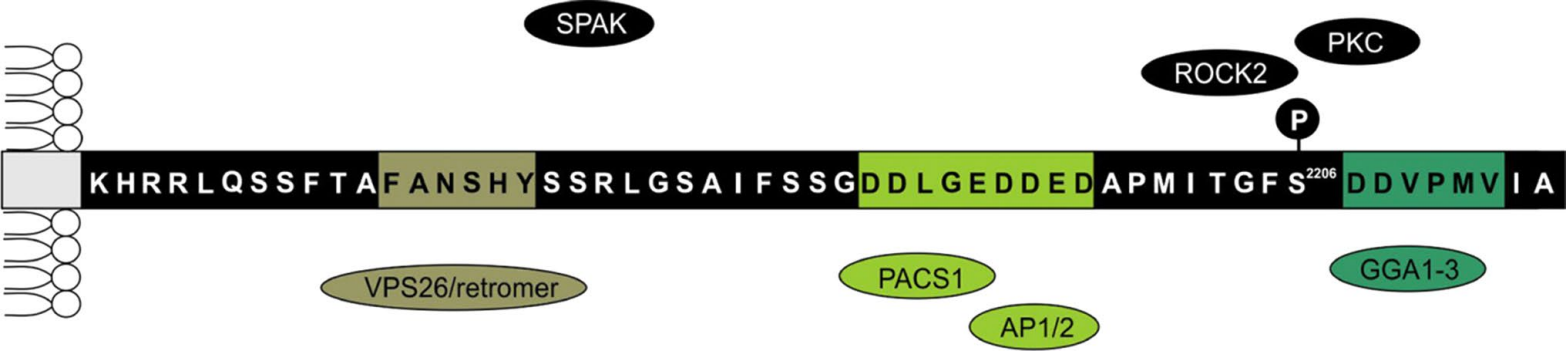
Protein interactions at the cytoplasmic domain of SORLA. The amino acid sequence of the cytoplasmic domain of human SORLA (Q92673, Uniprot) is shown. Binding sites for GGAs, PACS1, AP1 and 2 as well as for the VPS26 subunit of the retromer complex are *color* coded in *green*. Binding sites for SPAK, ROCK2, and PKC in the receptor tail are currently unknown. Amino acid residue serine 2206 is subject to phosphorylation by ROCK2

The second structural element with a decisive role in SORLA activity is the cytoplasmic tail of the receptor. This 54 amino acid domain harbors multiple motifs for protein–protein interaction and for post-translational modification (Fig. 4). Specifically, the tail includes binding sites for three cargo adaptor complexes, termed PACS1, GGA, and retromer that mediate the shuttling of SORLA between TGN and endosomes. Phosphofurin acidic cluster sorting protein 1 (PACS1) interacts with an acidic motif D^2190^DLGEDDED to mediate retrograde Golgi-to-endosome transport of the receptor [11, 85]. Retrograde sorting of SORLA is also governed by the retromer complex through binding of its VPS26 subunit to the motif F^2172^ANSHY in the receptor tail [26, 86, 87]. In contrast, anterograde sorting of SORLA is guided by the clathrin adaptors GGA1 and GGA2 (Golgi-localizing, γ-adaptin ear homology domain, ARF-interacting proteins) that bind to the D^2207^DVPMVIA element in the SORLA tail [36, 43, 85]. Adaptor protein (AP) 1 and 2 are tetrameric adaptor complexes that link cargo to the clathrin coat of endosomal and TGN vesicles. They also interact with the acidic motif in SORLA and possibly regulate endocytosis (AP2) and retrograde receptor sorting (AP1) [66]. Adaptor interactions are crucial for SORLA-dependent sorting and processing of APP, as deletion of individual adaptor-binding sites in the tail of SORLA causes the inability of the receptor to sort properly and results in aberrant routing and enhanced processing of APP in cells [11, 26, 36, 66, 85]. These distinct roles of anterograde versus retrograde sorting of SORLA have recently also been substantiated in vivo. Expression of mutant SORLA variants lacking the binding sites for retromer [23] or PACS1 [11] results in impaired retrograde routing of SORLA, and causes increased amyloidogenic processing of APP in the brain of transgenic mice. In contrast, in vivo disruption of the binding site for GGA blocks anterograde receptor sorting and reduces lysosomal catabolism of Aβ (and of SORLA) without impacting APP processing [23].

As well as directing receptor sorting, interactions at the tail of SORLA may also control receptor activities through signal transduction pathways, although the underlying concepts are not fully understood. Thus, binding of several kinases, including Ste-20-related proline-alanine-rich kinase (SPAK) [73], protein kinase C, as well as Rho-associated coiled-coil containing protein kinase (ROCK) 2 [37, 52] to the receptor tail has been documented. Phosphorylation of the cytoplasmic domain of SORLA at serine 2206 (possibly by ROCK2; Fig. 4) increases receptor activity and reduces APP processing [37].

## Functional implications of *SORL*1 gene variants in AD

Having defined the molecular basis of SORLA action, one can now start to appreciate how rare *SORL1* variants in the human population may influence the risk of sporadic or autosomal-dominant forms of AD. Obviously, many identified SNPs may not represent true functional variants, but be in linkage disequilibrium with yet unidentified sequence alterations. Still, the causal role of several SNPs in impacting SORLA expression and activity has been confirmed as detailed in the following.

Conceptually, non-coding SNPs may work through a change in *SORL1* transcription (Fig. 2). Little is known about the mechanisms that control expression of *SORL1* in cells, such as neurons. Typically, expression of *SORL1* is higher in proliferative cell types and decreases upon cellular differentiation as shown for neuroblastoma cells [38]. *SORL1* expression is controlled by DNA methylation [104] and by an enhancer element in exon 17 of the gene [10]. Several factors induce receptor expression, including hypoxia-inducible factor 1α [67], the omega-3 fatty acid DHA [57], and brain-derived neurotrophic factor (BDNF) [79]. Interestingly, neuronal expression of *SORL1* may not only be controlled at the level of gene transcription but also by alternative splicing. Full-length but also *SORL1* transcripts lacking exons 2 or 19 are found in the human brain. Levels of the transcript encoding the full-length receptor, but not those of the exon 2 deletion, are reduced in individuals with sporadic AD [29]. A long non-coding RNA that maps in an antisense direction to exon 1 in *SORL1* induces alternative splicing and reduces expression of the full-length receptor. Expression of this non-coding RNA is up-regulated in the AD brain and coincides with increased Aβ formation in cells [17].

Consistent with a suspected impact of some risk SNPs on *SORL1* transcription, Rogaeva and colleagues identified SNP#22-24 in the 3′ haplotype block (Fig. 2) to be associated with a 50 % reduction in mRNA levels in lymphoblasts of sporadic AD patients. Others showed association of *SORL1* mRNA levels with rs661057 in the 5′ gene region [29] or with the 5′ haplotype block in a cohort of healthy controls [59]. Sequence variations encoded by the 5′ haplotype block (SNPs #8-10; Fig. 2) result in loss of inducibility of *SORL1* by BDNF [103], while SNP#21 may impact splicing as predicted by in silico analysis [48]. Even some coding variants in *SORL1* may act by altering SORLA expression. For example, four coding sequence variants found in cases of early onset AD (p.D54 fs, p.G447 fs, p.W1216X, p.C1478X) are proposed to reduce transcript levels through nonsense-mediated RNA decay [65, 95]. A silent mutation encoded by SNP #19 (p.S1187=) reduces the efficiency of SORLA translation by changing from frequent to rare codon usage in the disease-associated minor allele [12]. Collectively, different mechanisms have been suggested to underlie the observed association of *SORL1* risk alleles with the production of functional transcripts. Why reduced expression of the receptor in some instances may cause late-onset, but in other cases early onset of AD remains a puzzling question that warrants further investigation.

**Fig. 5:**
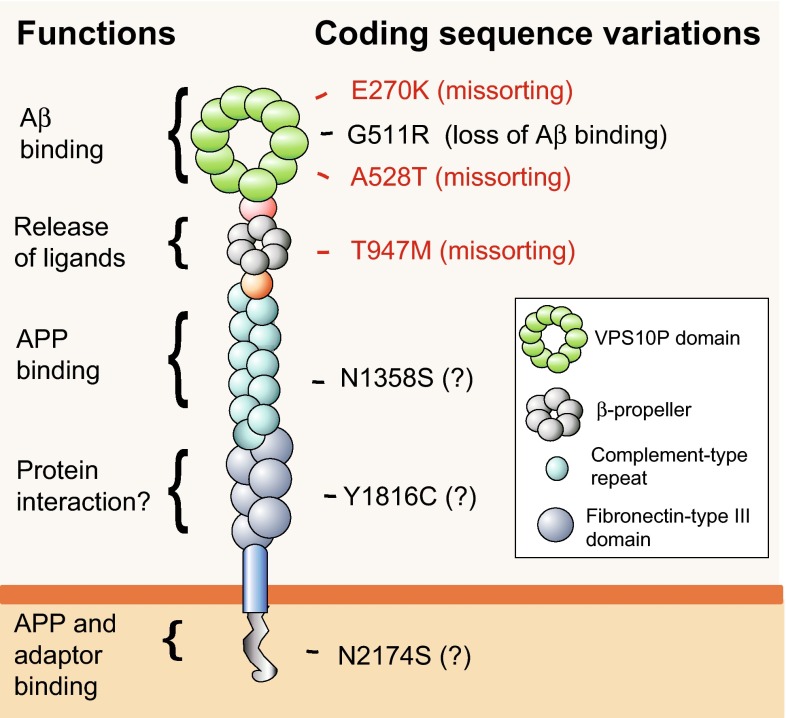
Functional modules in SORLA targeted by coding disease gene variants. Structural organization of the mature SORLA polypeptide indicating proposed functions for the various protein modules (to the *left*). Selected sequence variations identified in late- (*red*) or early onset (*black*) cases of AD are shown to the *right*. Mutations p.E270K, p.A528T, and p.T947M cause missorting of the receptor and APP ligand, increasing the extent of amyloidogenic processing [93]. Mutation p.G511R disrupts the binding site for Aβ, impairing SORLA-dependent lysosomal catabolism of this peptide [13]. Other domains in SORLA harboring AD-associated sequence alterations are the complement-type repeats, the fibronectin-type III domain, and the cytoplasmic tail as exemplified by the indicated coding variants [65, 95]. Whether these sequence variations also alter receptor functions is unresolved so far

Perhaps even more informative may be coding variants in *SORL1* that abrogate distinct receptor functions as they may help in further elucidating the molecular architecture of the receptor polypeptide (Fig. 5). Mutation p.G511R was identified in two affected individuals from a pedigree consistent with an autosomal-dominant mode of inheritance of AD [69]. This mutation disrupts the binding site for Aβ in the VPS10P domain of SORLA, resulting in the inability of the mutant receptor to facilitate lysosomal catabolism of Aβ [13]. Three additional coding variants found in cases of sporadic AD map to the VPS10P domain (p.E270K, p.A528T) or to the β-propeller (p.T947M; Fig. 5). All three receptor variants coincide with impaired retrograde sorting of APP and enhanced Aβ production when expressed in cells [93]. Additional SORLA coding sequence variants in early onset AD cases target the cluster of complement-type repeats, the fibronectin-type 3 domains, or the retromer recognition motif in the cytoplasmic receptor tail [65, 95]. Although no functional data are available as yet, these sequence alterations may disrupt APP or adaptor interaction with SORLA.

## SORLA, a target for diagnosis or treatment of AD?

SORLA is subject to proteolysis by metalloproteases, such as tumor necrosis factor-converting enzyme, resulting in shedding of the soluble ectodomain of the receptor [33, 35]. This soluble ectodomain can be detected in plasma and CSF. Ectodomain shedding disrupts the ability of SORLA to act as an intracellular sorting receptor but may serve to produce a soluble receptor fragment, termed soluble (s)SORLA, that acts as a signaling molecule. Such a function for sSORLA has recently been documented in bone morphogenetic protein signaling in adipose tissue [98]. While the relevance of sSORLA for AD-related processes still awaits clarification, the circulating levels of this fragment may provide an estimate of full-length receptor level or activity in brain tissue. Accordingly, several studies aimed at correlating sSORLA levels in CSF with brain pathology or with established biomarkers of AD, yet the results have been inconsistent so far. In some cohorts, the levels of sSORLA were significantly reduced in the lumbar samples of patients with mild to moderate probable AD as well as in ventricular CSF from autopsy-confirmed AD cases [56]. This observation would be in line with low levels of full-length SORLA being risk bearing. In contrast, others reported increased sSORLA levels in AD cases [40] or a positive association of sSORLA with BACE-1 activity [92] or sAPPβ and tau levels [3] in CSF of AD patients. As for these latter cases, a positive correlation of sSORLA levels with AD biomarkers may argue for enhanced ectodomain shedding as a pathological mechanism reducing the levels of active full-length receptor in the brain parenchyma.

While additional studies are warranted to substantiate sSORLA as a biomarker of AD, the therapeutic benefit of raising receptor levels to reduce the amyloidogenic burden is undisputed. As a proof of concept, increasing SORLA levels in the brain of transgenic mouse models has been shown to reduce Aβ levels, a mechanism attributed to the enhanced shunt of newly produced Aβ peptides into lysosomal catabolism in neurons [13]. Also, increasing brain SORLA levels by intracranial injection of BDNF has proven successful in reducing Aβ levels in mice [79]. An alternative strategy to strengthening the SORLA pathway may be provided by small molecules that stabilize the retromer complex. In cells, these molecular chaperones promote retrograde sorting of APP and decrease amyloidogenic processing, a mechanism that possibly works in a SORLA-dependent manner [60].

## Outlook

This review has focused on a role of SORLA in sorting of APP and Aβ to provide working models for a protective function of this receptor in AD. However, undoubtedly, this receptor is not specific to sorting of APP and Aβ, but has other protein targets as well. Of particular relevance to neurodegenerative processes, SORLA is able to sort several neurotrophin receptors, including the BDNF receptor TrkB [78], the receptor for glial cell-line-derived neurotrophic factor called GFRα1 [27], and the ciliary neurotrophic factor receptor α [54]. Thus, SORLA may represent a disease gene on which pathways in amyloidogenic processing and in trophic support of neurons converge. Obviously, further investigations are required to explore this intriguing concept, but they certainly offer the potential for exciting new insights into the genetic basis and pathological mechanisms of neurodegenerative disease.
